# The impact of alignment and scaling on biological inferences from landmark‐free morphometrics

**DOI:** 10.1111/joa.70227

**Published:** 2026-08-16

**Authors:** Lucy E. Roberts, Marco Camaiti, Anjali Goswami

**Affiliations:** ^1^ Natural History Museum London UK

**Keywords:** DAA, diffeomorphism, homology, interpretation, landmark‐free, methods, morphometrics, shape

## Abstract

Recent advances in shape quantification techniques have revolutionised the field of evolutionary morphology by providing a time‐efficient alternative to well‐established methods such as geometric morphometrics. In particular, landmark‐free methods are at the forefront of new developments in shape analysis, but concerns over their reproducibility and sensitivity to non‐biological variation have hindered more widespread adoption. A primary source of non‐biological bias in shape analysis is misalignment and incorrect scaling of specimens, which can result in capturing anatomically incongruent aspects of shape in otherwise homologous structures. Here, we compare methods for mesh alignment and scaling using a recently described landmark‐free approach, Deterministic Atlas Analysis (DAA). We find similarities between morphospaces derived from meshes aligned using most alignment protocols, but with large differences in variance structure. Surprisingly, the largest differences are not found between Procrustes‐based and non‐Procrustes‐based approaches, but between the Procrustes‐based method with internal scaling and all other alignment approaches. More broadly, scaling method has the greatest impact on shape variation captured by DAA. Based on these results, we make recommendations to ensure that alignment strategy maintains homology in landmark‐free morphometry. As in all morphometric analyses, violating principles of homology can result in spurious or inaccurate biological inferences in macroevolutionary studies of anatomy and must be explicitly considered in designing methodological frameworks.

## Graphical abstract

Landmark‐free morphometrics promises advancements in shape quantification for biology, but widespread adoption is hampered by concerns over reproducibility. We compare 3D mesh alignment and scaling protocols on a prominent landmark‐free approach, Deterministic Atlas Analysis, finding inconsistencies in centroid size distribution and variance structure. We make recommendations for explicitly incorporating homology into these procedures, ensuring biological signal is correctly inferred.

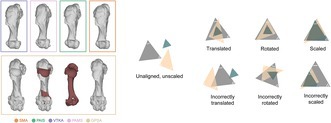

## 1 INTRODUCTION

Over the past several decades landmark‐based geometric morphometrics (GMM) has revolutionized our capacity to quantify and understand shape variation in the life sciences (Mitteroecker & Schaefer, 2022). 3D GMM in particular has enabled the study of complex anatomical structures, representing a well‐established method for capturing shape variation. Increasingly sophisticated methods have emerged to enhance the amount and quality of shape information that is captured; among these, sliding and surface semi‐landmarks are widely used for the high‐resolution sampling of shape (e.g. Ballell et al., 2022; Davies et al., 2024; Evers et al., 2022; Felice et al., 2021; Goswami et al., 2022; Huntley et al., 2021; Mennecart et al., 2022; Navalón et al., 2022; Sella‐Tunis et al., 2018; Tharakan et al., 2023). However, increases in the density of sampling also require greater computational demands, as well as an increase in the amount of time input required by researchers to complete landmarking tasks (Bardua et al., 2019; Goswami et al., 2019; Rummel et al., 2024). These factors, compounded with the rapidly increasing size of datasets, and proportional rates of user error in manual placement of landmarks, has limited the applications of standard GMM (Shearer et al., 2017).

A multitude of methods have been developed to reduce researcher input time, which can be broadly divided into two main approaches: automation of landmark placement (Devine et al., 2020; Porto et al., 2021; Porto & Voje, 2020) and landmark‐free shape quantification (Beg et al., 2005; Benn et al., 2019; Bône et al., 2018; Boyer et al., 2015; Godefroy et al., 2012; Imirzian et al., 2024; Lin et al., 2024; Pomidor et al., 2016; Thomas et al., 2023; Toussaint et al., 2021). Techniques to automatically place landmarks on surfaces use computer vision or deep‐learning approaches to position landmarks based on a template or training set (Devine et al., 2020; Porto et al., 2021; Porto & Voje, 2020). The downsides of these include the need for training datasets and relatively high computational costs (He et al., 2024). The performance of these methods relative to manual landmarking is context‐dependent; precision and accuracy can vary with anatomical complexity, taxonomic breadth and how easily homologous positions can be identified geometrically, that is based entirely on shape without user input (Devine et al., 2020; Percival et al., 2019; Szara et al., 2025).

Landmark‐free shape quantification entirely or nearly removes the need for landmarks by either utilising pseudo‐landmarks (e.g. Boyer et al., 2015; Porto et al., 2021), exploiting methods developed for mathematical function analysis (e.g. spherical harmonics; Abdallah et al., 2010; Gerig et al., 2001) and Fourier analysis (Godefroy et al., 2012), or otherwise registering diffeomorphic variations between geometric objects (e.g. Bône et al., 2018). Landmark‐free approaches have been successful at quantifying 2D variation (Mercan et al., 2013; Salili‐James et al., 2022), and an increasing number of studies are employing these approaches on 3D datasets (dos Reis et al., 2021; Imirzian et al., 2024; Macrì et al., 2019; Mulqueeney et al., 2025; Roberts et al., 2025; Sloan et al., 2024). Across all these approaches, anatomical structures are commonly represented as 3D meshes: digital surface models composed of interconnected vertices and faces that approximate geometry. Meshes can be derived by segmentation of tomographic images, which involves painting the region of interest in each 2D slice to build a 3D reconstruction of the element. 3D objects can also be reconstructed virtually through photogrammetry, where a 3D model is constructed from overlapping photographs (Evin et al., 2016; Giacomini et al., 2019; Muñoz‐Muñoz et al., 2016; Tsuboi et al., 2020), or using specialist 3D scanners that digitise the geometry, and sometimes the texture (colour), of an object using either structured light, laser or LIDAR technology (e.g. Artec 3D, n.d.; Bastir et al., 2019; Creaform 3D, n.d.; FARO, n.d.; Kalinowski et al., 2022).

**FIGURE 1:**
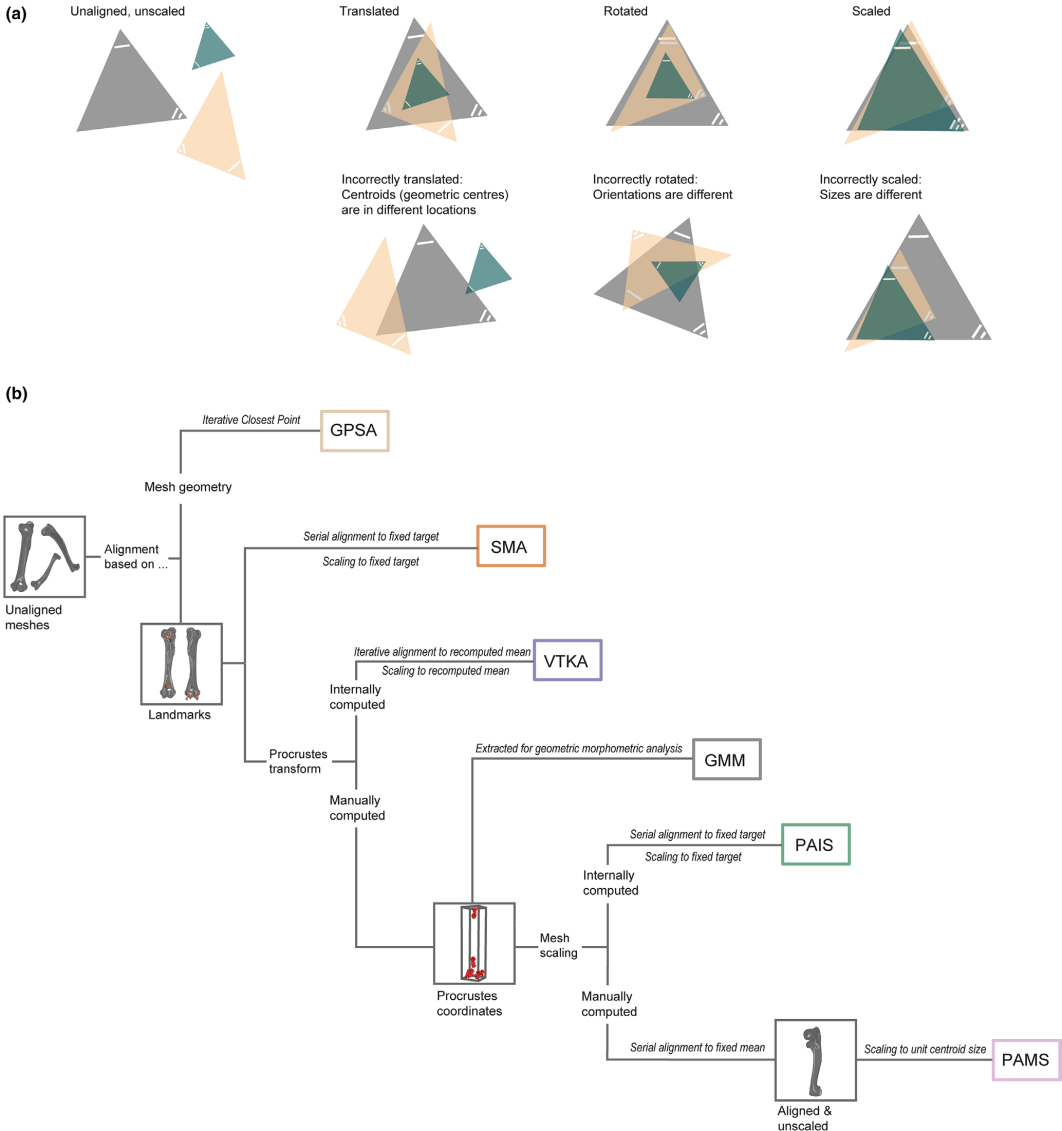
Descriptions of and differences between alignment protocols. (a) Schematic demonstrating alignment and scaling steps, alongside potential errors at each stage. (b) Flowchart describing the differences between methods compared herein.

Landmark‐based GMM extracts shape information as coordinate data. These coordinates are transformed using Generalised Procrustes Analysis (GPA), a standard technique for alignment of geometric morphometric data, in which landmarks are translated and rotated to the same position and scaled to unit centroid size (Figure 1a), where centroid size is defined as the square root of the summed squared distances of all landmarks from their geometric centre, or centroid (Rohlf & Slice, 1990). Manual landmarkers use knowledge of anatomy to place landmarks on homologous sites. Homology is defined as similarity of structures due to shared evolutionary origin, whereas the homology of landmark positions is a nuanced concept, encompassing the evolutionary definition, in addition to mathematical, anatomical, developmental and functional considerations, dependent on the precise research question (Wärmländer et al., 2019; Zelditch et al., 2004).

Concerns have been raised regarding landmark‐free morphometrics, particularly regarding the treatment of homology. Indeed, many landmark‐free methods have been called ‘homology‐free’ (Cardini, 2020; Klingenberg, 2008). Unlike landmark‐based methods where homology is explicitly encoded within the data (landmarks) themselves, in landmark‐free methods shape is quantified directly from anatomical surfaces. Consequently, alignment of the meshes themselves is critical in landmark‐free methods because it determines which anatomical regions are treated as corresponding between specimens. Previous research has shown that inconsistencies in geometry and topology can influence the outcomes of landmark‐free methods (Koehl & Hass, 2014, 2015). Meshes or images that are incorrectly aligned will create distributions in shape‐space that reflect non‐biological (or geometric) variation (Mulqueeney et al., 2025).

To address this issue previous studies of shape analysis have used a variety of methods to align meshes prior to landmark‐free shape analysis. Some of these methods involve alignment based on a small number of landmarks, such as with the *rotmesh.onto*() function in Morpho, in R (Mulqueeney et al., 2025; Roberts et al., 2025), or the Procrustes alignment function in VTK in Python (Imirzian et al., 2024). Others, such as Generalized Procrustes Surface Analysis (GPSA) offers a landmark‐free approach to mesh alignment (Pomidor et al., 2016). Despite these varying methods, and potentially higher sensitivity of landmark‐free morphometric methods to variation from errors in translation, rotation and scaling, no study has systematically compared how alignment and scaling methods influence the outcomes of landmark‐free morphometric analyses or assessed which methods best preserve biologically meaningful correspondence across a dataset. Here we use a dataset of 185 humeri of canids to compare five different methods for mesh alignment prior to landmark‐free analysis. These five approaches are as follows:

1. Alignment and scaling to a single reference mesh using *rotmesh.onto*() in Morpho (R), herein referred to as single‐mesh alignment (SMA)
2. Alignment and scaling to Procrustes‐transformed landmarks in *rotmesh.onto*() in Morpho (R), herein referred to as Procrustes alignment with internal scaling to reference mesh (PAIS)
3. Alignment to Procrustes‐transformed landmarks in *rotmesh.onto*() in Morpho (R), with separate scaling to a unit centroid size (R), herein referred to as Procrustes alignment with manual scaling (PAMS)
4. Procrustes alignment using the vtkProcrustesAlignmentFilter, in VTK (Python), herein referred to as VTK alignment (VTKA)
5. GPSA (General Procrustes Surface Analysis) alignment, herein referred to as GPSA

To compare these alignment protocols, we use Deterministic Atlas Analysis (DAA), a landmark‐free method that uses the outputs of Deformetrica (ver. 4.3.0) (Bône et al., 2018; Durrleman et al., 2014), processed using a pipeline developed by Toussaint et al. (2021) to extract shape variables. Deformetrica computes shape deformations (in 2‐ or 3D) in ambient space using large deformation diffeomorphic metric mapping (LDDMM) (Bône et al., 2018). In practice, this involves providing Deformetrica with a set of aligned meshes and a set of parameters including kernel type and kernel width, which determine how the deformations will be performed. Deformetrica computes a mean shape, applies control points and then computes the vector transformations from that mean shape to each provided mesh. The kernel is a function used to generate a vector field from the momenta; the kernel width determines the precision of the computed deformations, where a larger kernel width results in smoother deformations. In a practical sense, the kernel width determines the number of control points applied to the mean mesh. The computed deformations are non‐linear; the post‐processing steps written by Toussaint et al. (2021) enable the user to transform the outputs using non‐linear kernel principal component analysis (kPCA). These steps output kernel principal components, eigenvectors and eigenvalues, such that the user can visualise and use the computed shape variables in a fashion analogous to the analysis of results from a landmark‐based geometric morphometric analysis. In DAA, shape variables are derived from deformations of meshes from an atlas; therefore Procrustes superimposition must be conducted on the meshes themselves, rather than just on a set of landmarks.

In addition to these DAA analyses, we also conduct geometric morphometric analysis using the alignment landmarks to act as a comparative baseline. Our comparisons therefore include a sixth method:

6 Geometric morphometric analysis on the alignment landmarks, herein referred to as landmark‐based geometric morphometrics (GMM)

When comparing the resulting morphospaces, we hypothesise that those produced using PAMS and VTKA will be identical—both alignment methods use the same mathematical principles to align the meshes. We expect that these methods will also produce very similar results to landmark‐based GMM. We expect PAIS alignment to give very similar results, as only the scaling step differs mathematically from PAMS. We expect GPSA to produce results that are markedly different from VTKA, PAMS and PAIS—as homology is not explicitly accounted for during alignment. SMA is likely to produce results that appear biologically meaningful, but morphospaces are expected to differ from VTKA, PAMS and PAIS.

## 2 MATERIALS AND METHODS

### 2.1 Data collection

Our test dataset comprises 185 humeri of domestic dogs and their relatives across Canini. We produced 77 of these meshes using CT scans provided by the University of Liverpool Small Animal Teaching Hospital. We segmented the scans using the SPROUT toolkit (He et al., 2025) in Python (v3.10) to partially automate the process. Segmentations were cleaned and converted to .ply meshes in Avizo (Thermo Fisher Scientific, 2021). The remaining 108 meshes were produced by surface scanning specimens in museum collections (Table S1), using a Creaform Go! Scan 50. We processed the raw scans and converted them into .ply meshes using VXElements (ver. 2022.06.13). We made every mesh watertight and decimated down to 100,000 faces in Geomagic Wrap (3D Systems, 2017, ver. 2017.0.2.18). If meshes are not watertight deformetrica may interpret part of the inner surface or structure of those meshes as outer geometry, resulting in distorted mean shapes and deformations that do not represent the shape of the outer surfaces. We decimated every mesh to 100,000 faces to remove variation in surface complexity based on mesh resolution, Decimating to fewer faces will make deformetrica faster, but may compromise the shape of the mesh. The appropriate number of faces will depend on the size and shape of the structures under analysis, as well as the original resolution of the meshes. We recommend visually inspecting the effect of decimation to different numbers of faces on a subsample of the meshes under analysis to gauge the appropriate number for any particular analysis.

We applied seven landmarks to homologous positions (Figure S1) on each mesh in Stratovan Checkpoint (Stratovan Corporation, 2018), exported as .pts. files. Theoretically, as 3 points define a plane, this number should be adequate to align meshes, provided that the 3 points do not lie along a line. Additional points ensure that enough information is provided in all 3D for alignment. Here we use seven landmarks to remove the possible impact that too few landmarks may have on alignment. For all alignment methods based in R (ver. 4.3.2, R Core Team, 2014), we imported the meshes into the R Studio environment (ver. 2025.05.0 Posit team, 2025) using vcgPlyRead in Rvcg and the .pts. landmark files using read.pts. in Morpho (Schlager, 2017).

### 2.2 Alignment

Using the same meshes and landmarks (where landmarks are necessary) in each case, we ran five different processes to align meshes (Figure 1b):

#### 2.2.1 Single mesh alignment (SMA)

Following Mulqueeney et al. (2025) and Roberts et al. (2025) we used the *rotmesh.onto*() function in Morpho. We created a loop that sequentially rotates, reflects and scales each mesh to a chosen target landmark configuration. This function computes these transformations based on two sets of landmarks, a set pertaining to the mesh to be aligned, and a configuration pertaining to the target shape. Therefore, in addition to the raw meshes, we input the landmarks for each of these meshes, and a matrix containing only the landmarks of the target mesh: The first specimen in the list (Labrador 211,896). All meshes are rotated, reflected and scaled to this target. Scaling within *rotmesh.onto*() is done by applying the least‐squares scale factor that minimises Procrustes distances between the two landmark sets. Alignment and scaling to a different target mesh will change the scale and position of the whole set of meshes, such that a set of meshes aligned using Labrador 211,896 as the target will be differently aligned and scaled to a set using, for example, Greyhound 225,226 as the target, but the shape differences measured within the two sets should not differ.

#### 2.2.2 Procrustes alignment with internal scaling (PAIS)

We modified the SMA method, using Procrustes‐transformed landmarks to align meshes instead of a raw reference shape. We used *rotmesh.onto*(), but instead of aligning each mesh to a single individual landmark configuration, we transformed the landmarks with Procrustes superimposition using *gpagen*() in geomorph and sequentially aligned each mesh to its corresponding Procrustes configuration. *rotmesh.onto*() has a logical ‘scale’ argument which we set to TRUE for this alignment method. Internally *rotmesh.onto*() uses the *rotonto*() function which rotates, translates and optionally scales matrices to minimise the sums of squares. Importantly, when scaling using *rotmesh.onto*() the target configuration is scaled to minimise squared distances relative to the reference.

#### 2.2.3 Procrustes alignment with manual scaling (PAMS)

To apply Procrustes superimposition to the meshes, including scaling to a unit centroid size, we first used *rotmesh.onto*() with scale set to FALSE, such that the meshes are rotated and translated to the Procrustes mean (‘consensus’ shape in geomorph). We then calculated the centroid size of each mesh using *cSize*() in Morpho. Then we scaled each mesh using *scalemesh*() in Morpho which scales the vertices of an input mesh by a chosen scalar, in this case 1/*cSize*() for each mesh. This method ensures that the alignment process occurs in the same order as a Procrustes superimposition: translation, rotation and then scaling/dilation (Mitteroecker et al., 2013), and the same order of operations performed by *rotmesh.onto*() (Schlager, 2017).

#### 2.2.4 VTK alignment (VTKA)

Following Imirzian et al. (2024), we aligned the meshes using *vtkProcrustesAlignmentFilter* in VTK. This conducts a GPA following Gower (1975). This algorithm works by calculating a mean configuration of the landmarks, aligning each landmark set to this mean and registering each mesh according to the transformation from the original set to the set aligned with the mean shape. It then proceeds iteratively, recomputing a new mean shape and re‐aligning to this mean, unlike SMA, PAIS and PAMS that proceed serially using a fixed target. In VTKA this process repeats until the mean stabilises. Scaling within vtkLandmarkTransform is done by applying the ratio of root‐mean‐square centroid sizes between the target and source landmark sets, computed as part of a similarity transformation that minimises the least‐squares alignment error.

#### 2.2.5 GPSA

General Procrustes Surface Analysis is a full landmark‐free method for shape analysis that utilises the Iterative Closest Point (ICP) algorithm to align a set of meshes and calculate final distance metrics (Pomidor et al., 2016). GPSA associates each point on a mean surface to its geometrically similar point on all of the other surfaces. The algorithm transforms the surfaces, seeking to minimise the distances between all the pairs of points by rotating and translating. This process is repeated until no further improvement can be made. One of the outputs of GPSA is the aligned meshes, which we use herein. GPSA does not perform scaling as part of its ICP‐based superimposition; instead, size is removed as a preprocessing step by scaling each mesh to unit centroid size prior to superimposition.

### 2.3 Deterministic atlas analysis

After alignment we converted each set of aligned meshes to .vtk format where necessary (Schroeder et al., 2006); meshes aligned using VTKA have been converted pre‐alignment. For each of the five alignment methods we ran Deterministic Atlas Analysis (DAA) in Deformetrica (Bône et al., 2018; Durrleman et al., 2014), on a terminal in the Ubuntu operating system (ver. 22.04.4 LTS). Deformetrica creates an atlas, or mean shape, from the input meshes, applies control points to this atlas and then computes the vector transformations required to warp that atlas to each of the original meshes. There are several model parameters chosen by the user, including the kernel width, noise and the number of time points or intermediate warps between the atlas and each mesh. We chose a noise parameter of 1.0, a maximum of 150 iterations and 20 time points, which are the number of warping iterations conducted between the atlas and each mesh, following Mulqueeney et al. (2025) and Roberts et al. (2025). The kernel width determines the number of control points Deformetrica places on the atlas. We used approximately 500 control points for each analysis. The number of control points cannot be directly controlled, but is instead determined by the kernel width parameter. A lower kernel width results in a larger number of control points, such that the deformations captured are smaller in scale, but may introduce noise at large quantities. The number of control points resulting from a particular kernel width parameter depends on the size of the atlas, and therefore the size of the input meshes. For this reason, different methods of scaling require different kernel widths to achieve the same approximate number of control points. The kernel widths used herein range from 0.056 to 12.4 mm, resulting in between 476 and 560 control points (Table S2).

The outputs of Deformetrica are the intermediate and final warps from the atlas to each mean shape, the atlas shape itself, the control point coordinates and a set of momenta, which represent the vector transformations required from each control point to warp the mesh into each final shape. The momenta are nonlinear data, meaning that a traditional principal components analysis is inappropriate. We used a modified version of the post‐processing pipeline developed by Toussaint et al. (2021), which implements kernel principal component analysis (kPCA) of the momenta, using the scikit‐learn (Pedregosa et al., 2011) module in Python. In kPCA a kernel function is used to project the data into a high‐dimensional feature space, enabling decomposition into linear principal components (Schölkopf et al., 1997). Following this we exported the kernel Principal Components (kPCs) and transferred them into the R Studio Environment for visualisation.

### 2.4 Geometric morphometric analysis

To create a comparative baseline for the kPC morphospaces, we conducted geometric morphometric analysis on the alignment landmarks. We Procrustes‐transformed the landmarks using *gpagen*(), removing differences due to translation and rotation and scaling to unit centroid size. We then conducted a principal components analysis using *gm,prcomp*() on the Procrustes coordinates.

### 2.5 Comparing alignment methods

Across the different alignment methods, axes of variation may vary in polarity (arbitrarily) and they may constitute different aspects and proportions of total variation; for example, the shape variation captured by PC2 in an analysis using PAIS may correspond to that of PC3 using PAMS. Before comparing methods, to remove the impact of arbitrary differences in sign, we coerced the direction of each principal component using the principal components derived from the fixed alignment landmarks as a reference. To assess differences in the order of (k)PCs due to different variance structures, we established the PCs derived from the fixed landmarks as a reference and computed the correlation between this reference and every other set of principal components. We then used the *solve_LSAP*() (linear sum assignment problem) function in the *clue* package (Hornik, 2005) to find the optimal one‐to‐one pairing of columns such that total correlation across each pair of sets of principal components is maximised. Following this, we reordered each kPC table according to these maximised correlations to examine the proportion of variance captured by each equivalent component. We compared the original kPC indexes to the reordered values by calculating Kendall's rank correlation, where a higher coefficient is indicative of greater congruence between original and reordered indexes. We assessed whether different alignment procedures preserve the same specimen‐level relationships using partial least squares regression (PLS), using the *two.b.pls*() function in *geomorph*. We extract r‐PLS, the correlation coefficient from each PLS, as a metric representing the degree of correlation between the two sets of (k)PCs. This coefficient can be between 0 and 1, where values closer to 1 signify stronger correlation that is if the shape captured by two methods is identical, we would recover an r‐PLS of 1.

We evaluated sensitivity to alignment method by conducting a series of PERMANOVA (permutational multivariate analyses of variance) tests using the *adonis2*() function in *vegan*, on kPC scores, assessing alignment method and breed/species as marginal effects. To investigate differences in scaling between alignment methods, we calculated the centroid size of all meshes aligned using each method and the centroid size of the Procrustes‐transformed landmarks for comparison. We logged and scaled these sets of centroid sizes such that the values could be compared directly.

## 3 RESULTS

### 3.1 Shape variation amongst Canini

**FIGURE 2:**
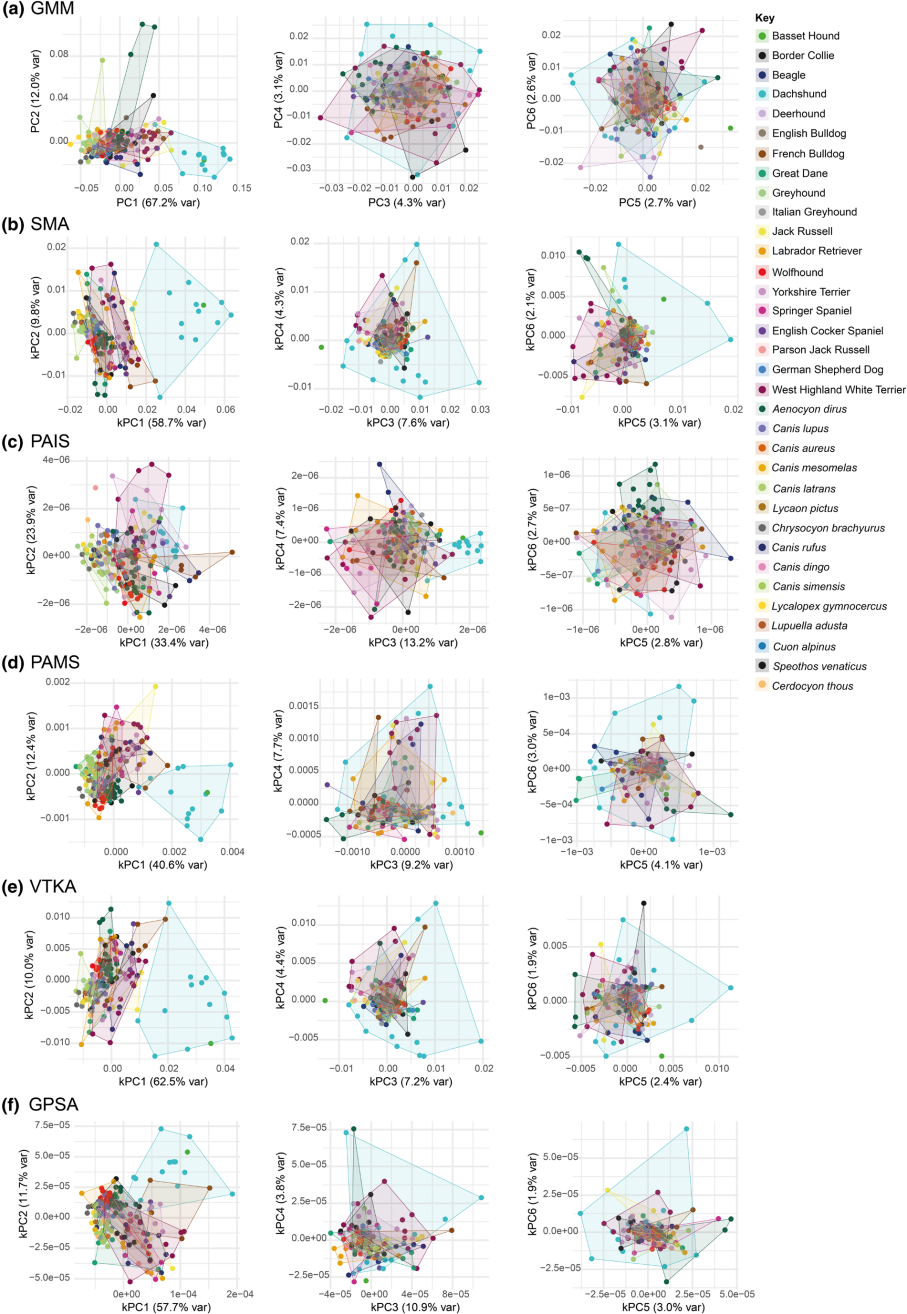
Shape variables derived following different alignment protocols. (a) Landmark‐based 3D GMM. (b) DAA after SMA alignment. (c) DAA after PAIS alignment. (d) DAA after PAMS alignment. (e) DAA after VTKA alignment. (f) DAA after GPSA alignment.

Most alignment and scaling procedures, and GMM, show similar patterns of variation amongst canid humeri. Amongst all wild dogs and most domestic breeds little variation in shape is apparent, evidenced by substantial overlap of convex hulls in most morphospaces, particularly those representing the majority of shape variation in each analysis (i.e. PC1 and PC2) (Figure 2, Figure S2). Dachshunds, separate markedly from other species and breeds, either across PC1 (GMM, SMA, PAMS, VTKA) or PC1 and PC2 (GPSA).

We recover superficial similarity between most morphospaces constructed from identical Deterministic Atlas Analyses of the same set of meshes aligned using different methods. Among the dataset, dachshunds have a markedly distinct humeral shape and are therefore expected to separate from all other breeds and species under analysis in, at minimum, the (k)PC1‐2 morphospaces. This is the case for all but one of the (k)PC1‐2 morphospaces (Figure 2). Results derived from meshes aligned using Procrustes Alignment in R and the internal scaling (PAIS) recover little to no difference in the shape of dachshund humeri relative to other species and breeds of domestic dog (Figure 2c). Morphologies represented by the extremes of each (k)PC differ between PAIS and every other method; kPC represents an axis between the extreme dachshund morphology at high values of (k)PC1 and a more elongate shape with less pronounced articulation surfaces at low values of (k)PC1 (Figure S2). In the analysis based on PAIS‐aligned meshes kPC1 does represent variation between more and less elongate elements but fails to capture the morphological extremes of the dataset.

PERMANOVAs run on each set of (k)PCs recover a significant relationship between breed and humeral shape for each set of shape variables, except for the analyses using PAIS (Table 1), suggesting that alignment using PAIS is obscuring biological variation. A PERMANOVA assessing total shape variation captured by the first 10 principal components against alignment method, however, does not recover a significant difference in total shape variation captured using the different alignment methods (*p* = 1). Pairwise PERMANOVAs also indicate no significant differences (*p* = 1 for all pairs).

**TABLE 1:** Results of PERMANOVA analyses testing if humeral shape differs significantly between breeds.

Method	Rsq	*p* value
GMM	0.67	0.001
SMA	0.625	0.001
PAIS	−0.47	1
PAMS	0.525	0.001
VTKA	0.644	0.001
GPSA	0.404	0.001

**FIGURE 3:**
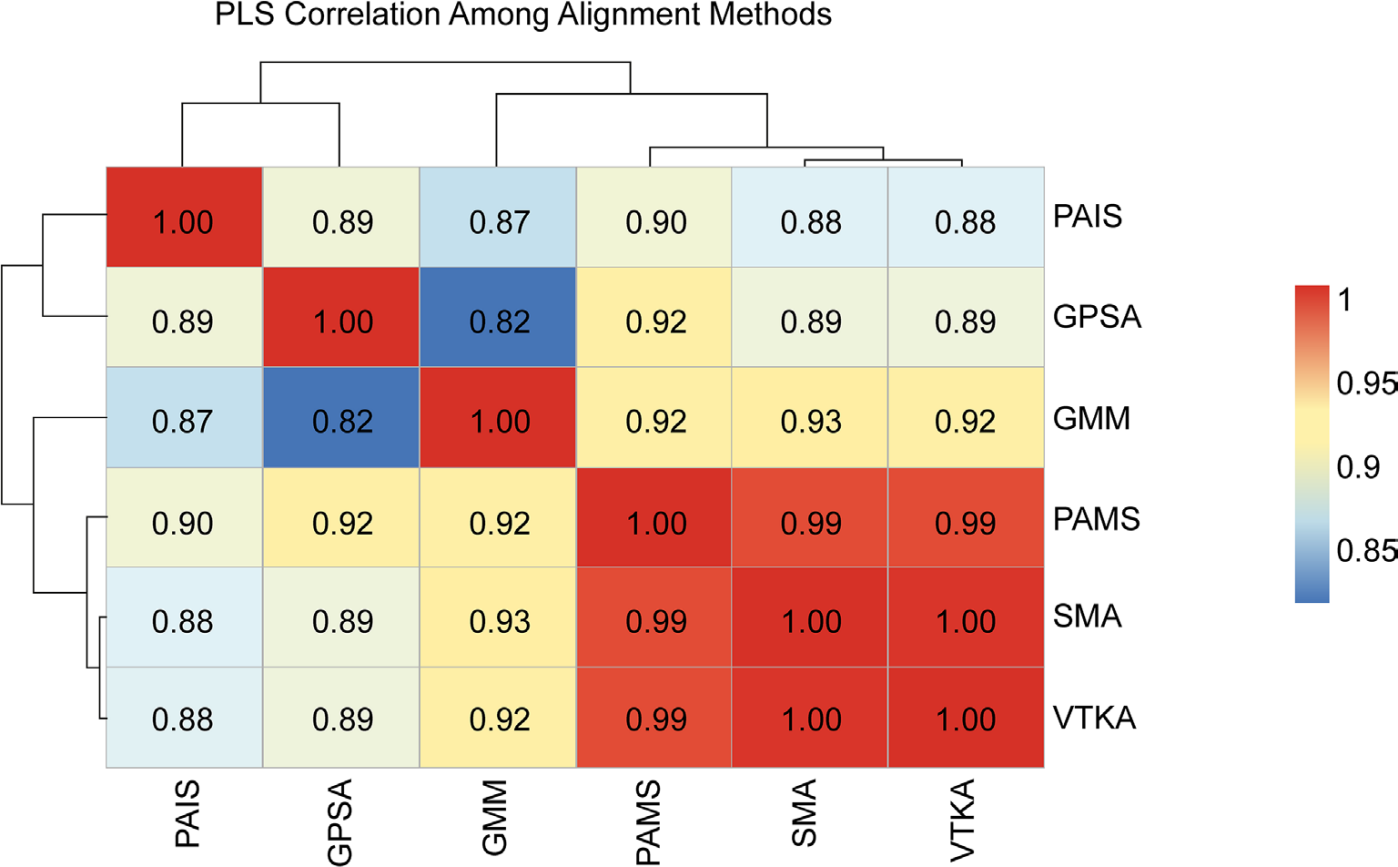
Partial Least Squares correlations between the first 10 shape variables derived using different alignment methods. *p* < 0.001 for all correlations.

Partial least squares analyses demonstrate that the total shape variation captured by each method is statistically equivalent; every method, including traditional GMM using the alignment landmarks (GMM), is highly and strongly correlated; all pairwise tests recover significant relationships (Figure 3). Comparisons between results derived using PAMS, SMA and VTKA result in almost perfect correlations (r‐PLS = 0.99–1). PAIS‐based results are less strongly correlated (r‐PLS = 0.87), which is consistent with observed differences in PAIS‐based morphospaces. Traditional GMM on the alignment landmarks (GMM) is less strongly correlated with GPSA‐based shape variation, while still maintaining an r‐PLS of 0.82.

### 3.2 Scaling and alignment

**FIGURE 4:**
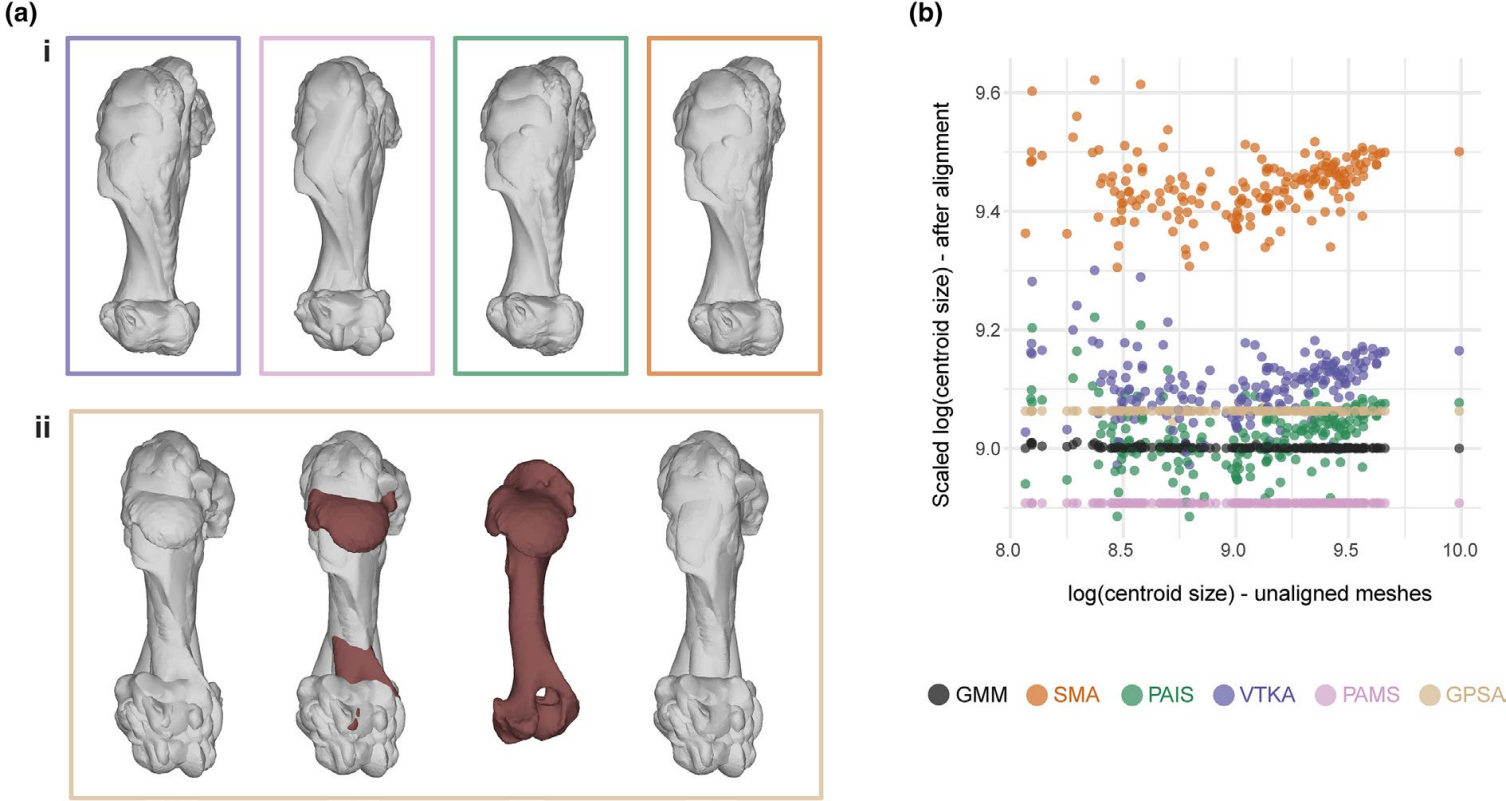
Differences in mesh position, rotation and size following different alignment and scaling protocols. (a) All meshes aligned using (i) VTKA, PAMS, PAIS and SMA and (ii) GPSA, alongside the misaligned mesh and all meshes without the misaligned individual. (b) Scaled, logged centroid size of all aligned meshes and Procrustes‐transformed alignment landmarks (GMM) against logged centroid size of the original, unaligned meshes.

Visual inspection of the aligned meshes reveals superficial similarity of alignment between every set except for the GPSA‐aligned meshes. In all other sets every humerus is aligned to the same sagittal, dorsal and transverse planes (Figure 4a). Each humeral head and condyle face in the same direction and the proximal and distal ends of each humerus are aligned. In the set of GPSA‐aligned meshes, however, one mesh is incorrectly aligned craniocaudally: Specimen 257,098 is rotated approximately 180° about the proximodorsal axis (Figure 4a,ii). In addition to this, there are visible differences in the position and rotation of meshes aligned using GPSA compared to those aligned using different methods—this is particularly apparent at the distal end of the humerus; the lateral and medial condyles of GPSA‐aligned meshes visibly do not align proximodistally. GPSA proceeds solely by geometric similarity, with no user‐specified information of biological homology. Here, this results in meshes that are either only roughly aligned or are incorrectly aligned in at least one axis (Figure 4a,ii).

For each set of aligned meshes we extracted the centroid sizes and logged and scaled the values to aid comparison, plotting the values against the logged centroid size of the original, unaligned meshes (Figure 4b). For comparison we also include the logged, scaled centroid sizes of the Procrustes‐transformed alignment landmarks. PAMS and GPSA produce sets of aligned meshes that have consistent centroid sizes, indicated by invariance on the y‐axis (Figure 4b), similar to Procrustes transformation of landmarks. Centroid sizes of meshes aligned and scaled using VTKA, PAIS and SMA, however, produce aligned meshes with markedly variable centroid sizes, particularly amongst originally smaller meshes, indicated by y‐axis variation (Figure 4b). Size variation between meshes is modified in VTKA, PAIS and SMA, but it is not removed. This modified scaling will influence shape variables derived from these meshes, meaning that such variables will not describe pure shape transformation, which should be without influence of translation, rotation and size.

### 3.3 Variance structure

**FIGURE 5:**
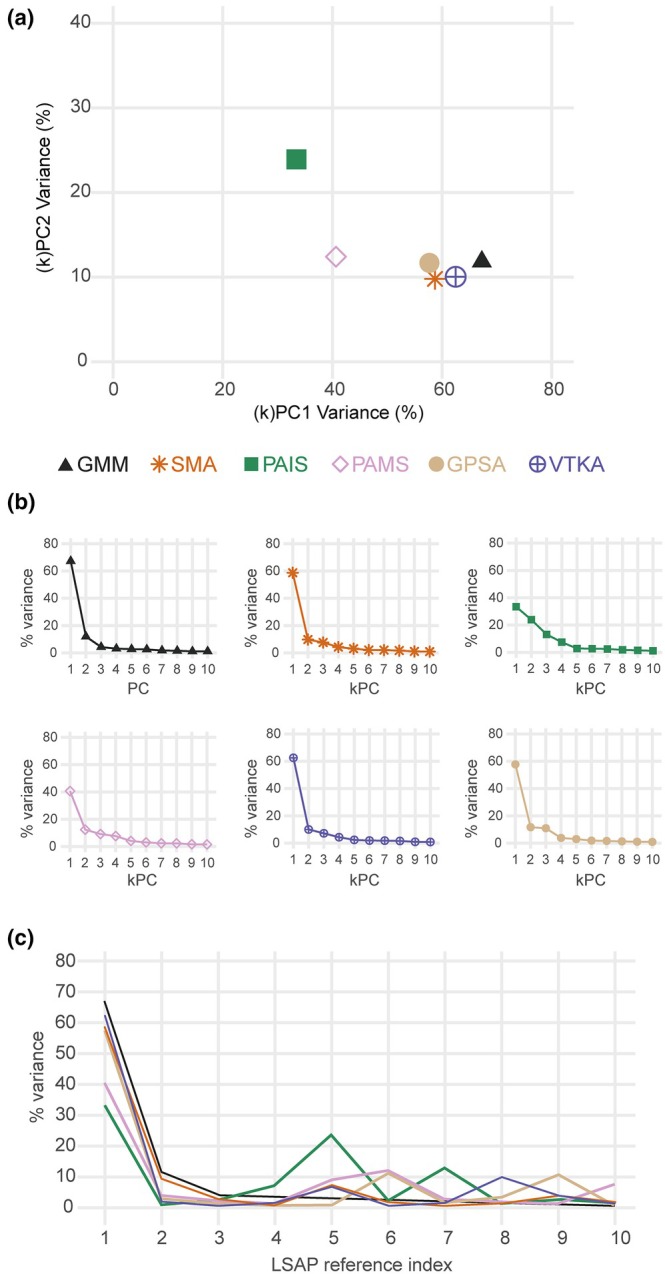
Differences in variance structure between shape variables derived using different alignment methods. (a) Proportion of variance encompassed by the first two principal components for every method. (b) Proportion of variance encompassed by the first 10 principal components for each method. (c) Proportion of variance reordered according to correlation between axes.

The proportion of variance encompassed by the first two principal components varies between methods. GMM on the alignment landmarks results in PC1 encompassing 67% of total variance, PC2 encompassing 12% and each subsequent principal component encompassing less than 5% total variation (Figure 5a,b). All DAA‐based analyses result in kernel PC1s encompassing less than 67%. kPC1 derived from meshes aligned using GPSA, VTKA and SMA range between 58% and 62% of total variation, kPC1 derived from PAMS‐aligned meshes encompasses 40% and from PAIS is just 33%. Optimal matching of (k)PCs reveals substantial redistribution of explained variance across corresponding axes (Figure 5c, Table 2). The first principal component, however, does not change under any alignment method. Rank correlations between the original indexes and the indexes reordered following optimal matching using *solve_LSAP*() show that VTKA and GPSA alignment result in the most substantial divergence (corr = 0.16) followed by PAIS and PAMS (0.29) and SMA (3.8) (Table 2).

**TABLE 2:** Ordering of kPCs following correlation of PC axes and reordering according to LSAP. Principal components derived from GMM of alignment landmarks are used as the reference order. Rank cor refers to Kendall's rank correlation between the original and reordered index.

GMM (reference)		SMA		PAIS		PAMS		VTKA		GPSA	
LSAP index	% var	LSAP index	% var	LSAP index	% var	LSAP index	% var	LSAP index	% var	LSAP index	% var
1	67.25	1	58.71	1	33.43	1	40.64	1	62.48	1	57.69
2	11.97	2	9.79	10	1.14	5	4.12	5	2.39	5	2.98
3	4.31	5	3.09	6	2.73	7	2.41	9	0.98	7	1.61
4	3.13	9	1.11	4	7.45	9	1.69	6	1.93	9	1.05
5	2.75	3	7.64	2	23.92	3	9.18	3	7.22	8	1.29
6	2.59	7	2.07	7	2.48	2	12.41	10	0.87	2	11.68
7	1.77	10	0.97	3	13.18	6	3.05	7	1.85	6	1.89
8	1.58	8	1.72	9	1.5	8	2.38	2	10.04	4	3.83
9	1.17	4	4.26	5	2.85	10	1.58	4	4.36	3	10.94
10	1.16	6	2.1	8	1.83	4	7.71	8	1.62	10	0.92
	Rank cor	0.38		0.29		0.29		0.16		0.16	

## 4 DISCUSSION

As new methods emerge it is critical to develop an understanding of the appropriateness and sensitivity of methodological choices, to ensure that assumptions are robust and that interpretations are biologically grounded. If our quantification of anatomy is inaccurate or skewed, we risk making similarly inaccurate or skewed inferences. This has the potential to impact our understanding of the relationship between anatomy and macroevolution, function and development. We find that the results of landmark‐free DAA derived from meshes aligned using different methods are largely similar, such as the clear distinction between dachshund humeral shape and that of all other breeds. In nearly all (k)PC1‐(k)PC2 morphospaces, dachshunds separate from all other breeds and species; the sole exception is the kPC1‐kPC2 morphospace derived from PAIS aligned meshes (Procrustes Alignment, Internal Scaling), across which dachshunds do not separate from other breeds or species (Figure 2). Despite this, the total shape variation quantified in each case is comparable. PERMANOVA across (k)PCs in each method shows no significant difference in total shape variation represented by the first 10 (k)PCs captured by each method. Partial Least Squares further demonstrates that total shape variation captured in each run is commensurate among the various alignment methods (Figure 3). Variance structure, however, differs between the analyses (Figure 5). Therefore, observed differences in morphospaces are largely driven by redistribution of variance across the principal components, rather than changes in the underlying multivariate structure of the data.

Differences among distributions of variance across the components can be explained by variation in the features detected by each shape quantification method. The key distinction between landmark‐based and landmark‐free methods lies in the specification of the features to be extracted. While in GMM, homologous features to be compared are generally intentionally and manually selected by the user, in landmark‐free approaches morphology is computed from the geometry of the whole structure instead, capturing more of the morphology but being unable to prioritise between more or less biologically‐salient features. In landmark‐based GMM, homology is built into the coordinate data itself—whereas in DAA the vector transformations that are used to derive shape variables do not in and of themselves encode homology. Therefore, this information needs to be incorporated explicitly via structure/specimen selection and alignment.

Here, we held the approach to capturing shape variation constant and focused on a more foundational decision in morphometrics: how to align specimens. Each alignment method produced commensurable results with regards to total shape (Figure 3, Table 1); analyses using multiple kPCs as multivariate data may therefore see little difference in the results of downstream analyses when employing any alignment protocol, with the exception of PAIS. However, eigenvalue structure varies between protocols (Figure 5). Redistribution of variance across PC axes means that interpretations based on individual components, dimensionality truncation or variance‐weighted distances will be affected by the alignment method. As a consequence, evolutionary interpretations that are based only on a few principal components will vary more widely based on method, even when the underlying variation captured across the whole shape space remains constant. As indicated by Uyeda et al. (2015), this should caution against the use of only a few PC axes for evolutionary modelling. However, given that the number of axes is specified a priori in these methods, the partitioning of variance structure can be redistributed for commensurate comparison (Schölkopf et al., 1997; Toussaint et al., 2021).

DAA computes shape deformations based on differences in the location of corresponding control points applied to the surface of each mesh under analysis. If the alignment of these meshes fails to properly incorporate homology, the resulting deformations will likewise fail to preserve homological integrity and biological relevance. Prior works have discussed retaining homology when employing landmark‐free methods by isolating individual elements rather than analysing composite structures (e.g. constituent parts of the skull rather than the whole skull) (Mulqueeney et al., 2025). Here we demonstrate that even when homology is not considered during alignment—that is landmark‐free alignment based purely on geometric similarity (GPSA)—superficially sensible and realistic results can be obtained (Figure 2, Figure 3). Previous studies have assessed the efficacy of DAA by analysing the distance between resulting kernel principal coordinates and PCs derived using traditional, landmark‐based GMM (Mulqueeney et al., 2025). Here we show that similar morphospaces to landmark‐based GMM can result from incorrectly aligned (GPSA, Figure 4a,ii) or scaled (PAIS, VTKA, SMA, Figures 2 and 4b) meshes. If the underlying mathematics of methods used to derive shape variables is incorrect, biologically meaningful inferences cannot be confidently drawn, which has the potential to impact the interpretation of anatomical comparisons within a macroevolutionary context.

Our recommendation concluded from this work, to ensure that meshes are correctly aligned and scaled such that shape variables derived using landmark‐free morphometrics uphold principles of homology, is to employ PAMS. PAMS conducts a global Procrustes alignment of the meshes, aligning each mesh to the Procrustes mean shape, and scaling to unit centroid size. GPSA commonly results in mesh misalignment (Figure 4a,ii) and PAIS, VTKA, SMA result in asymmetrically scaled meshes. While Procrustes transformation should result in uniform scaling of the whole dataset, PAIS, VTKA, SMA do not (Figure 4b). The marked difference in morphospaces derived using PAMS and PAIS demonstrates the critical nature of correctly accounting for scale during mesh alignment (Figure 2c,d). DAA can also be conducted in 2D; although not tested explicitly herein, the PAMS alignment protocol should also ensure biological correspondence in a 2D pipeline.

Future studies should consider consolidating alignment and shape analysis, such that users can input meshes and conduct landmark‐free shape analysis in a pipeline that explicitly accounts for homology, rather than the input of such analyses to be pre‐aligned meshes. Furthermore, future studies should consider these recommendations in light of other landmark‐free morphometric methods. For example, Dense Correspondence Analysis (Rolfe & Maga, 2023) implements an ICP algorithm for alignment; GPSA alignment also uses ICP and results in the misalignment of specimens (Figure 4a).

To conclude, our comparison of alignment methodologies for landmark‐free methods highlight that, while DAA is robust to alignment choices regarding the total shape variation captured, the variance distribution across shape space is sensitive to the translation, rotation and scaling of meshes. Alignment protocols that do not consider homology or properly account for scale can influence eigenvalue structure. Evolutionary and comparative analyses that employ variance‐weighting or truncation of principal components may suffer from the conflation of biological signal with methodological artefacts. Therefore, we emphasise the necessity to include explicit biological information in the alignment and scaling steps (i.e. employing PAMS) prior to DAA, or any other landmark‐free morphometric method that uses pre‐aligned meshes to be able to test biologically meaningful hypotheses. These findings highlight the need for integrated pipelines in landmark‐free morphometrics to ensure interpretability, reproducibility and confidence in biologically meaningful results.

## AUTHOR CONTRIBUTIONS

Study design: LER, MC; Data analysis: LER; Figures: LER, MC; Manuscript writing and editing: LER, MC, AG.

## Supporting information


**Figure S1.** Landmarks used for alignment and comparative 3D geometric morphometrics.
**Figure S2.** Shape variation along (k)PC1 and (k)PC2 axes, represented by individuals at the extremes of each axis for each analysis.
**Table S1.** Specimen and scan information. Scan source codes refer to: Natural History Museum, London, UK (NHMUK); Rancho La Brea, Los Angeles, USA (La Brea); Smithsonian National Museum of Natural History, Washington DC, USA (USNM); Yale Peabody Museum, New Haven, USA (YPM); and University of Liverpool Small Animal Teaching Hospital, Liverpool UK (UoL_SATH).
**Table S2.** Parameters used in each Deformetrica analysis.

## Data Availability

All code is publicly available on github repository https://github.com/LucyEmmaRoberts/landmark‐free. Meshes and landmark data are publicly available on figshare https://doi.org/10.6084/m9.figshare.33145403.
