# Supplementary material for: The impact of alignment and scaling on biological inferences from landmark‐free morphometrics
Source: J Anat. 2026 Aug 16:10.1111/joa.70227. Online ahead of print. doi: 10.1111/joa.70227 (PMC13478407; doi:10.1111/joa.70227)
Supplement: Supplementary file 1 [file JOA-9999-0-s001.docx]

Supplementary material for: The impact of alignment and scaling on biological inferences from landmark-free morphometrics

Lucy E. Roberts^*1^, Marco Camaiti^1^ & Anjali Goswami^1^

*Corresponding author: lucy.roberts@nhm.ac.uk

1. Natural History Museum, Cromwell Road, London, SW7 5BD

Figure S1: Landmarks used for alignment and comparative 3D geometric morphometrics

Figure S2: Shape variation along (k)PC1 and (k)PC2 axes, represented by individuals at the extremes of each axis for each analysis.

Table S1: Specimen and scan information. Scan source codes refer to: Natural History Museum, London, UK (NHMUK); Rancho La Brea, Los Angeles, USA (La Brea); Smithsonian National Museum of Natural History, Washington DC, USA (USNM); Yale Peabody Museum, New Haven, USA (YPM); and University of Liverpool Small Animal Teaching Hospital, Liverpool UK (UoL_SATH)

| **Specimen number** | **Scan modality** | **Scan source** | **Breed** |
| --- | --- | --- | --- |
| 10767 | Surface | La Brea | *Aenocyon dirus* |
| 10771 | Surface | La Brea | *Aenocyon dirus* |
| 10776 | Surface | La Brea | *Aenocyon dirus* |
| 5669 | Surface | La Brea | *Aenocyon dirus* |
| 5675 | Surface | La Brea | *Aenocyon dirus* |
| 5705 | Surface | La Brea | *Aenocyon dirus* |
| 5747 | Surface | La Brea | *Aenocyon dirus* |
| 5753 | Surface | La Brea | *Aenocyon dirus* |
| 5756 | Surface | La Brea | *Aenocyon dirus* |
| 5758 | Surface | La Brea | *Aenocyon dirus* |
| 5760 | Surface | La Brea | *Aenocyon dirus* |
| 5767 | Surface | La Brea | *Aenocyon dirus* |
| 5773 | Surface | La Brea | *Aenocyon dirus* |
| 5775 | Surface | La Brea | *Aenocyon dirus* |
| 5779 | Surface | La Brea | *Aenocyon dirus* |
| 5782 | Surface | La Brea | *Aenocyon dirus* |
| 5787 | Surface | La Brea | *Aenocyon dirus* |
| 5791 | Surface | La Brea | *Aenocyon dirus* |
| 5799 | Surface | La Brea | *Aenocyon dirus* |
| 5802 | Surface | La Brea | *Aenocyon dirus* |
| 4943/2611 | Surface | YPM | Basset Hound |
| 232558 | CT | UoL_SATH | Beagle |
| 252396 | CT | UoL_SATH | Beagle |
| 253642 | CT | UoL_SATH | Beagle |
| 254861 | CT | UoL_SATH | Beagle |
| 257932 | CT | UoL_SATH | Beagle |
| 258140 | CT | UoL_SATH | Beagle |
| 238163 | CT | UoL_SATH | Border Collie |
| 242343 | CT | UoL_SATH | Border Collie |
| 247832 | CT | UoL_SATH | Border Collie |
| 250882 | CT | UoL_SATH | Border Collie |
| 255119 | CT | UoL_SATH | Border Collie |
| 257415 | CT | UoL_SATH | Border Collie |
| 816 | Surface | NHMUK | *Canis aureus* |
| 1952.4.9.2 | Surface | NHMUK | *Canis dingo* |
| 1868.4.14.1 | Surface | NHMUK | *Canis dingo* |
| 1954.11.15.1 | Surface | NHMUK | *Canis dingo* |
| 9657 | Surface | La Brea | *Canis latrans* |
| 9662 | Surface | La Brea | *Canis latrans* |
| 9665 | Surface | La Brea | *Canis latrans* |
| 9667 | Surface | La Brea | *Canis latrans* |
| 9668 | Surface | La Brea | *Canis latrans* |
| 9669 | Surface | La Brea | *Canis latrans* |
| 9671 | Surface | La Brea | *Canis latrans* |
| 9672 | Surface | La Brea | *Canis latrans* |
| 9674 | Surface | La Brea | *Canis latrans* |
| 9679 | Surface | La Brea | *Canis latrans* |
| 9680 | Surface | La Brea | *Canis latrans* |
| 9682 | Surface | La Brea | *Canis latrans* |
| 9686 | Surface | La Brea | *Canis latrans* |
| 9688 | Surface | La Brea | *Canis latrans* |
| 9693 | Surface | La Brea | *Canis latrans* |
| 9694 | Surface | La Brea | *Canis latrans* |
| 9797 | Surface | La Brea | *Canis latrans* |
| 13412.31861 | Surface | NHMUK | *Canis latrans* |
| 1987.1 | Surface | NHMUK | *Canis latrans* |
| 9655 | Surface | La Brea | *Canis latrans* |
| 9660 | Surface | La Brea | *Canis latrans* |
| 118023 | Surface | La Brea | *Canis latrans* |
| 15287 | Surface | YPM | *Canis lupus* |
| 1936.11201 | Surface | NHMUK | *Canis lupus* |
| 1937.2102 | Surface | NHMUK | *Canis lupus* |
| 33.294 | Surface | YPM | *Canis lupus* |
| 55.5.14.11 | Surface | NHMUK | *Canis lupus* |
| 5786 | Surface | La Brea | *Canis lupus* |
| 5820 | Surface | La Brea | *Canis lupus* |
| 6386 | Surface | La Brea | *Canis lupus* |
| 9659 | Surface | La Brea | *Canis lupus* |
| 84.1312 | Surface | NHMUK | *Canis lupus* |
| 86.1595 | Surface | NHMUK | *Canis lupus* |
| 1991.586 | Surface | NHMUK | *Canis mesomelas* |
| 1410.4055 | Surface | YPM | *Canis rufus* |
| 274080 | Surface | USNM | *Canis rufus* |
| 274107 | Surface | USNM | *Canis rufus* |
| 306291 | Surface | USNM | *Cerdocyon thous* |
| 338981 | Surface | USNM | *Cerdocyon thous* |
| 1958.12.11.1 | Surface | NHMUK | *Chrysocyon brachyurus* |
| 586580 | Surface | USNM | *Chrysocyon brachyurus* |
| 86.211 | Surface | NHMUK | Cocker Spaniel |
| 1952.4.7.10 | Surface | NHMUK | Cocker Spaniel |
| 258985 | Surface | USNM | *Cuon alpinus* |
| 587829 | Surface | USNM | *Cuon alpinus* |
| 74.882 | Surface | NHMUK | Dachshund |
| 74.883 | Surface | NHMUK | Dachshund |
| 22194 | Surface | USNM | Dachshund |
| 213979 | CT | UoL_SATH | Dachshund |
| 216192 | CT | UoL_SATH | Dachshund |
| 228420 | CT | UoL_SATH | Dachshund |
| 237465 | CT | UoL_SATH | Dachshund |
| 253589 | CT | UoL_SATH | Dachshund |
| 254590 | CT | UoL_SATH | Dachshund |
| 257010 | CT | UoL_SATH | Dachshund |
| 257098 | CT | UoL_SATH | Dachshund |
| 257593 | CT | UoL_SATH | Dachshund |
| 258467 | CT | UoL_SATH | Dachshund |
| 238168 | Surface | USNM | Deerhound |
| 243799 | CT | UoL_SATH | Deerhound |
| 6835/922 | Surface | YPM | English Bulldog |
| 220351 | CT | UoL_SATH | English Springer Spaniel |
| 224384 | CT | UoL_SATH | English Springer Spaniel |
| 238211 | CT | UoL_SATH | English Springer Spaniel |
| 243506 | CT | UoL_SATH | English Springer Spaniel |
| 250188 | CT | UoL_SATH | English Springer Spaniel |
| 612231 | Surface | USNM | English Springer Spaniel |
| 9501/3886 | Surface | YPM | English Springer Spaniel |
| 241035 | CT | UoL_SATH | French Bulldog |
| 245805 | CT | UoL_SATH | French Bulldog |
| 246762 | CT | UoL_SATH | French Bulldog |
| 255544 | CT | UoL_SATH | French Bulldog |
| 256334 | CT | UoL_SATH | French Bulldog |
| 255074 | CT | UoL_SATH | German Shepherd Dog |
| 4925/2595 | Surface | YPM | German Shepherd Dog |
| 4934/2596 | Surface | YPM | German Shepherd Dog |
| 4937/4006 | Surface | YPM | German Shepherd Dog |
| 8004/2683 | Surface | YPM | German Shepherd Dog |
| 231994 | CT | UoL_SATH | Great Dane |
| 235560 | CT | UoL_SATH | Great Dane |
| 238920 | CT | UoL_SATH | Great Dane |
| 247384 | CT | UoL_SATH | Great Dane |
| 251908 | CT | UoL_SATH | Great Dane |
| 145_72.898 | Surface | NHMUK | Great Dane |
| 7251/1362 | Surface | YPM | Great Dane |
| 1995.212 | Surface | NHMUK | Greyhound |
| 225226 | CT | UoL_SATH | Greyhound |
| 261035 | Surface | USNM | Greyhound |
| 200000 | CT | UoL_SATH | Greyhound |
| 53.80.2522 | Surface | NHMUK | Greyhound |
| A22308 | Surface | USNM | Greyhound |
| A22690 | Surface | USNM | Greyhound |
| A34805/A25880 | Surface | USNM | Greyhound |
| A38235/020805 | Surface | USNM | Greyhound |
| 35140 | Surface | USNM | Italian Greyhound |
| 213129 | CT | UoL_SATH | Jack Russell |
| 246210 | CT | UoL_SATH | Jack Russell |
| 249958 | CT | UoL_SATH | Jack Russell |
| 250612 | CT | UoL_SATH | Jack Russell |
| 222431 | CT | UoL_SATH | Labrador |
| 228257 | CT | UoL_SATH | Labrador |
| 231670 | CT | UoL_SATH | Labrador |
| 236773 | CT | UoL_SATH | Labrador |
| 244195 | CT | UoL_SATH | Labrador |
| 253627 | CT | UoL_SATH | Labrador |
| 253675 | CT | UoL_SATH | Labrador |
| 258701 | CT | UoL_SATH | Labrador |
| 1940.1.20.15 | Surface | NHMUK | Lupulella adusta |
| 92141 | Surface | USNM | *Lycalopex gymnocercus* |
| 92143 | Surface | USNM | *Lycalopex gymnocercus* |
| 1948.3302 | Surface | NHMUK | *Lycaon pictus* |
| 2669.4301 | Surface | YPM | *Lycaon pictus* |
| 2670.4301 | Surface | YPM | *Lycaon pictus* |
| 247962 | CT | UoL_SATH | Parson Jack Russell |
| 270368 | Surface | USNM | *Speothos venaticus* |
| 270370 | Surface | USNM | *Speothos venaticus* |
| 211043 | CT | UoL_SATH | West Highland White Terrier |
| 220019 | CT | UoL_SATH | West Highland White Terrier |
| 227260 | CT | UoL_SATH | West Highland White Terrier |
| 239804 | CT | UoL_SATH | West Highland White Terrier |
| 244390 | CT | UoL_SATH | West Highland White Terrier |
| 247854 | CT | UoL_SATH | West Highland White Terrier |
| 248812 | CT | UoL_SATH | West Highland White Terrier |
| 249127 | CT | UoL_SATH | West Highland White Terrier |
| 250482 | CT | UoL_SATH | West Highland White Terrier |
| 251048 | CT | UoL_SATH | West Highland White Terrier |
| 257571 | CT | UoL_SATH | West Highland White Terrier |
| 259056 | CT | UoL_SATH | West Highland White Terrier |
| 238046 | CT | UoL_SATH | Wolfhound |
| 289976 | Surface | USNM | Wolfhound |
| 347480 | Surface | USNM | Wolfhound |
| 350000 | Surface | USNM | Wolfhound |
| 396991 | Surface | USNM | Wolfhound |
| 398536 | Surface | USNM | Wolfhound |
| 398908 | Surface | USNM | Wolfhound |
| 399049 | Surface | USNM | Wolfhound |
| 399050 | Surface | USNM | Wolfhound |
| 506600 | Surface | USNM | Wolfhound |
| 518126 | Surface | USNM | Wolfhound |
| 534281 | Surface | USNM | Wolfhound |
| 7987/2666 | Surface | YPM | Wolfhound |
| 220373 | CT | UoL_SATH | Yorkshire Terrier |
| 238845 | CT | UoL_SATH | Yorkshire Terrier |
| 239179 | CT | UoL_SATH | Yorkshire Terrier |
| 246781 | CT | UoL_SATH | Yorkshire Terrier |
| 257703 | CT | UoL_SATH | Yorkshire Terrier |
| 257704 | CT | UoL_SATH | Yorkshire Terrier |
| 259364 | CT | UoL_SATH | Yorkshire Terrier |

Table S2: Parameters used in each Deformetrica analysis.

| Alignment method | Control points | Kernel width (mm) |
| --- | --- | --- |
| SMA | 480 | 18 |
| PAIS | 476 | 0.057 |
| PAMS | 504 | 1 |
| VTKA | 480 | 12.4 |
| GPSA | 560 | 0.227 |
